# Targeted multi-platform metabolome analysis and enzyme activity analysis of kiwifruit during postharvest ripening

**DOI:** 10.3389/fpls.2023.1120166

**Published:** 2023-03-07

**Authors:** Jipeng Mao, Zhu Gao, Mengfei Lin, Xiaoli Zhang, Xinyi Ning, Xuchen Gong, Yupeng Lu, Lu Chen, Xiaoling Wang

**Affiliations:** ^1^ Institute of Biological Resources, Jiangxi Academy of Sciences, Nanchang, Jiangxi, China; ^2^ Jinggangshan Institute of Biotechnology, Jiangxi Academy of Sciences, Ji’an, Jiangxi, China; ^3^ College of Environmental and Chemical Engineering, Nanchang Hangkong University, Nanchang, Jiangxi, China

**Keywords:** kiwifruit, soluble sugar, organic acid, energy metabolite, hormone, enzyme activity

## Abstract

Kiwifruit is a climacteric fruit, in which the accumulation of flavor substances mainly occurs at the postharvest ripening stage. However, the dynamic changes in metabolite composition remain poorly understood. Here, targeted multi-platform metabolome analysis based on GC-MS and UPLC-MS/MS and enzyme activity analysis were performed at different postharvest ripening stages of kiwifruit. A total of 12 soluble sugars and 31 organic acids were identified. The main soluble sugars are sucrose, glucose and fructose, which exhibited similar variation tendencies along with the extension of ripening. The main organic acids are citric acid, quinic acid and malic acid, which showed different variation patterns. A total of 48 energy metabolites were identified, which were classified into two groups based on the content variation. The content of substances related to the respiratory metabolic pathway decreased gradually along with postharvest ripening, and there was obvious accumulation of downstream products such as amino acids at the late ripening stage. A total of 35 endogenous hormones were identified, among which seven cytokinins were highly accumulated at the later stage of softening. We further investigated the dynamic changes in the activities of 28 ripening-related enzymes. As a result, the activities of 13 enzymes were highly correlated with changes in starch, total pectin, and soluble sugars, and those of seven enzymes were closely associated with the change in firmness. In conclusion, this study comprehensively describes the dynamic changes in soluble sugars, organic acids, hormones, energy substances, and ripening-related enzyme activities during kiwifruit postharvest ripening, and provides a theoretical basis for the postharvest quality improvement of kiwifruit.

## Introduction

Kiwifruit (*Actinidia* spp.) is an economically important and popular fresh fruit originating from China and currently cultivated globally (Huang et al., 2013). Approximately 52 species and 21 varieties of kiwifruit have been recognized, all of which are functionally dioecious (Sutherland et al., 2017; Cozzolinoa et al., 2020). Kiwifruit is highly appreciated for its unique flavor and high contents of vitamins, dietary fiber, lipids, amino acids, flavonoids, minerals, and other nutrient components recognized as beneficial to humans (Hu et al., 2014; Wang et al., 2022). *A. deliciosa* and *A. chinensis* are two most commercially important species (Folletta et al., 2019). With the constant improvement of cross breeding and cultivation management technologies, the yield of kiwifruit has been rapidly increasing. However, the huge postharvest loss and not ‘ready-to-eat’ have been prominent problems hindering the development of the kiwifruit industry.

Kiwifruit is generally regarded as a climacteric fruit, but has a different ripening process from typical climacteric fruits such as tomato and banana. The initiation of softening, ethylene production, and climacteric are temporally separated during kiwifruit ripening (Chen et al., 2021). The ripening process of kiwifruit can be divided in three phases: slow softening phase I, with high firmness, starch content and production of little volatiles; rapid softening phase II, with a rapid reduction of firmness to about 20% of the initial value; ethylene-dependent phase III, with the production of internal ethylene and characteristic volatiles (Atkinson et al., 2011). Kiwifruit is usually harvested under an unripe condition, and the developmental stage at which the fruit is harvested greatly determines its postharvest ripening process and storability (MacRae et al., 1989; Mack et al., 2017). In addition, the accumulation of flavor substances and the increase in edibility mostly occur during postharvest ripening.

Fruit flavor is largely determined by soluble sugars, organic acids, and volatile compounds. The accumulation of soluble sugars increases with the degradation of starch during kiwifruit postharvest ripening. α-amylase (α-Amy), β-amylase (β-Amy), starch phosphorylase (SP), starch debranching enzymes (SDBE), sucrose synthase (SUS), sucrose phosphate synthetase (SPS), acid invertases (AI), neutral invertases (NI), hexokinase (HK), and phosphofructokinase (PFK) have been reported to be involved in sugar production (Fung et al., 2003; Richardson et al., 2004; Rolland et al., 2006; Zeeman et al., 2010; Moscatello et al., 2011; Nardozza et al., 2013; Hu et al., 2016). Firmness is an intuitive index to measure the degree of softening, which is mainly determined by changes in cell wall structure and composition, particularly pectin, hemicellulose and cellulose. Polygalacturonase (PG), pectin methylesterase (PME), cellulase (CE), xyloglucan endotransglycosylase (XET), α-arabinfuranosidease (α-AF), β-glucosidase (β-GLU), and β-galactosidase (β-GAL) have been demonstrated to be involved in these changes (Redgwell and Fry, 1993; Brummell et al., 2004; Huang et al., 2020). Different fruits have various compositions and contents of organic acids. Previous studies have revealed that quinic acid, citric acid, and malic acid are main organic acids in kiwifruit (Nishiyama et al., 2008), and the tricarboxylic acid (TCA) cycle is responsible for the metabolism of citric and malic acids (Kou et al., 2018). Volatile compounds are another class of compounds contributing to fruit flavor. Wang et al. (2022) identified 28 volatiles in ‘Hongyang’ kiwifruit through metabonomics, and further studied their changes and regulatory mechanisms during fruit development and ripening. In addition, fruit ripening also involves plant endogenous hormones such as ethylene, auxin, methyl jasmonate, and melationin (Li et al., 2014; Jabbar and East, 2016; Li et al., 2017; Liu et al., 2020; Li et al., 2021). Therefore, it is important to comprehensively understand the changes in metabolites during kiwifruit postharvest ripening.

Metabolomics is an important part of systems biology that can be used to identify and quantify endogenous small-molecule metabolites in organisms or biofluid samples (Kaiser, 2012; Tian et al., 2021). In this study, targeted multi-platform analysis of the metabolome based on gas chromatography-mass spectrometry (GC-MS) and ultrahigh liquid chromatography-tandem mass spectrometry (UPLC-MS/MS) was performed to investigate the complex changes in metabolites in kiwifruit during postharvest ripening. The findings are expected to improve the theoretical basis for the development of postharvest technologies of kiwifruit.

## Materials and methods

### Plant materials and measurements

Kiwifruit (*Actinidia chinensis* cv. Jinyan) samples were collected from Fengxin County Doctor Kiwifruit Base (E114°45’, N28°34’), Yichun City, Jiangxi Province, China. Fruit with uniform size were harvested at 180 d after pollination and immediately transported to Jiangxi Academy of Science, which were then stored at 20 °C and sampled from 0 to 12 d to represent different ripening stages. The flesh free of skin and seeds was cut into small pieces, rapidly frozen in liquid nitrogen and then stored at –80 °C for further metabolome and enzyme activity analysis. Three biological replicates were performed for each experiment, with each replicate consisting of 12 fruit.

Fruit firmness was measured at two equatorial regions of peeled (approximately 1 mm) fruit using the GY-4 fruit firmness tester (AIPLI, Zhejiang, China) fitted with an 8-mm plunger and GY-4 holder. Soluble solid concentration (SSC) was measured using a digital hand-held refractometer (Atago, Tokyo, Japan). The content of titratable acid was measured using the standard method (GB/T 8210-2011, China). Starch and total pectin content were determined using the assay kits (starch: CN.AKSUO15C, total pectin: CN. AKSU069M, Beijing Boxbio Science & Technology, Co., Ltd, China), following the instruction of the previously described method. A total of 28 enzymes related to ripening were determined in this study, and all enzymes were extracted under ice-bath and detected by corresponding assay kits (**Supplementary Table 1**).

### Extraction and determination of soluble sugars by GC-MS

Soluble sugars were extracted and analyzed according to the methods described previously with some modifications (Medeiros and Simoneit, 2007; Sun et al., 2016; Zhao et al., 2019). Freeze-dried materials were crushed using a mixer mill (MM 400, Retsch) with a zirconia bead for 1.5 min at 30 Hz. About 20 mg of powder was diluted to 500 μL with methanol: isopropanol: water (3: 3: 2, v/v/v), vortexed for 3 min and ultrasound treated for 30 min. The extract was centrifuged at 14,000 rpm under 4°C for 3 min. About 50 μL of the supernatant was mixed with 20 μL internal standard (ribitol, 100 μg/mL) and evaporated under nitrogen gas stream. The evaporated samples were transferred to the lyophilizer for freeze drying. The residue was used for the further derivatization as follows: the sample was mixed with 100 μL solution of methoxyamine hydrochloride in pyridine (15 mg/mL). The mixture was incubated at 37°C for 2 h. Then, 100 μL of BSTFA was added into the mixture and kept at 37°C for 30 min after vortex mixing. The mixture was analyzed by GC-MS after dilution to an appropriate concentration.

Agilent 7890 gas chromatograph coupled to a 5975 C mass spectrometer with a DB-5MS column (30 mm length × 0.25 mm i.d. × 0.25 μm film thickness, J&W Scientific, USA) was employed for GC-MS analysis of soluble sugars. Helium was used as the carrier gas at a flow rate of 1 mL/min. Injection was made in the splitless mode and the injection volume was 2 μL. The oven temperature was held at 70°C for 1 min, then raised to 112°C at 30°C/min and held for 3 min, raised to 175°C at 15°C/min and held for 1 min, raised to 190°C at 3°C/min and held for 2 min, raised to 240°C at 35°C/min, raised to 280°C at 10°C/min and held at the temperature for 2.5 min. All samples were analyzed in the selective ion monitoring mode. The ion source and transfer line temperature were 230°C and 240°C, respectively.

### Extraction and determination of organic acids, endogenous hormones, energy metabolites by UPLC-MS/MS

After the sample was thawed and smashed, 0.10 g of the sample was mixed with 1 mL of 70% methanol/water. The sample was vortexed for 3 min at 2500 r/min and centrifuged at 12000 r/min for 10 min at 4°C. About 600 μL of supernatant was taken and put into a new centrifuge tube and then placed in –20°C refrigerator for 30 min, Then, the supernatant was centrifuged again at 12000 r/min for 10 min at 4°C. After centrifugation, 200 μL of supernatant was used for organic acid determination directly, and another 200 μL of supernatant was passed through Protein Precipitation Plate for energy metabolite determination.

Endogenous hormones were extracted according to previously described method (Li et al., 2016a). Frozen kiwifruit sample (50 mg) was ground into powder (30 Hz, 1 min), and then dissolved in 1 mL methanol/water/formic acid (15:4:1, v/v/v). Then, 10 μL internal standard mixed solution (100 ng/mL) was added into the extract as internal standard (IS) for the quantification. The mixture was vortexed for 10 min, and then centrifuged for 5 min (12000 r/min, and 4°C). The supernatant was transferred to clean plastic microtubes, followed by evaporation to dryness and dissolved in 100 μL 80% methanol (V/V), and filtered through a 0.22 μm membrane filter for endogenous hormone determination.

The sample extracts were detected using a UPLC-ESI-MS/MS system. The analytical conditions were as follows: for organic acid, ACQUITY HSS T3 (2.1×100 mm, 1.8 μm) column; solvent system, water 0.05% formic acid (A), acetonitrile with 0.05% formic acid (B). The gradient was started at 5% B (0 min), increased to 95% B (8-9.5 min), finally ramped back to 5% B (9.6-12 min); flow rate, 0.35 mL/min; temperature, 40°C; injection volume: 2 μL. For energy metabolites, ACQUITY UPLC BEH Amide (2.1 × 100 mm, 1.7 μm); solvent system, water with 10mM Ammonium acetate and 0.3% Ammonium hydroxide (A), 90% acetonitrile/water 27(v/v) (B). The gradient was started at 95% B (0–1.2 min), decreased to 70% B (8 min),50% B (9–11 min), finally ramped back to 95% B (11.1–15 min); flow rate, 0.4 mL/min; temperature, 40°C; injection volume: 2 μL. For endogenous hormones, Waters ACQUITY UPLC HSS T3 C18 (2.1 × 100 mm, 1.8 µm); solvent system, water with 0.04% acetic acid (A), acetonitrile with 0.04% acetic acid (B); gradient program, started at 5% B (0-1 min), increased to 95% B (1–8 min), 95% B (8–9 min), finally ramped back to 5% B (9.1–12 min); flow rate, 0.35 mL/min; temperature, 40°C; injection volume, 2 μL.

Linear ion trap (LIT) and triple quadrupole (QQQ) scans were acquired on a triple quadrupole-linear ion trap mass spectrometer (QTRAP), QTRAP^®^ 6500+ LC-MS/MS System, equipped with an ESI Turbo IonSpray interface, which was operated in both positive and negative ion modes and controlled by Analyst 1.6.3 software. The ESI source operation parameters were as follows: ion source, ESI+/-; source temperature 550°C; ion spray voltage (IS) 5500 V(Positive), –4500 V (Negative); curtain gas (CUR) was set at 35 psi, respectively.

### Data collection and statistic analysis

Based on the self-established metabolite database MWDB (Metware database) and the public metabolite information database, the primary and secondary mass spectrometry data were qualitatively analyzed. The soluble sugars, organic acids, and endogenous hormones were analyzed using scheduled multiple reaction monitoring (MRM). Data acquisition was performed using Analyst 1.6.3 software (Sciex). Multiquant 3.0.3 software (Sciex) was used to quantify all metabolites. Pearson’s correlation analysis was performed using the R software.

## Results

### Fruit firmness, SSC, titratable acid, starch and total pectin content during postharvest ripening

**Figure 1:**
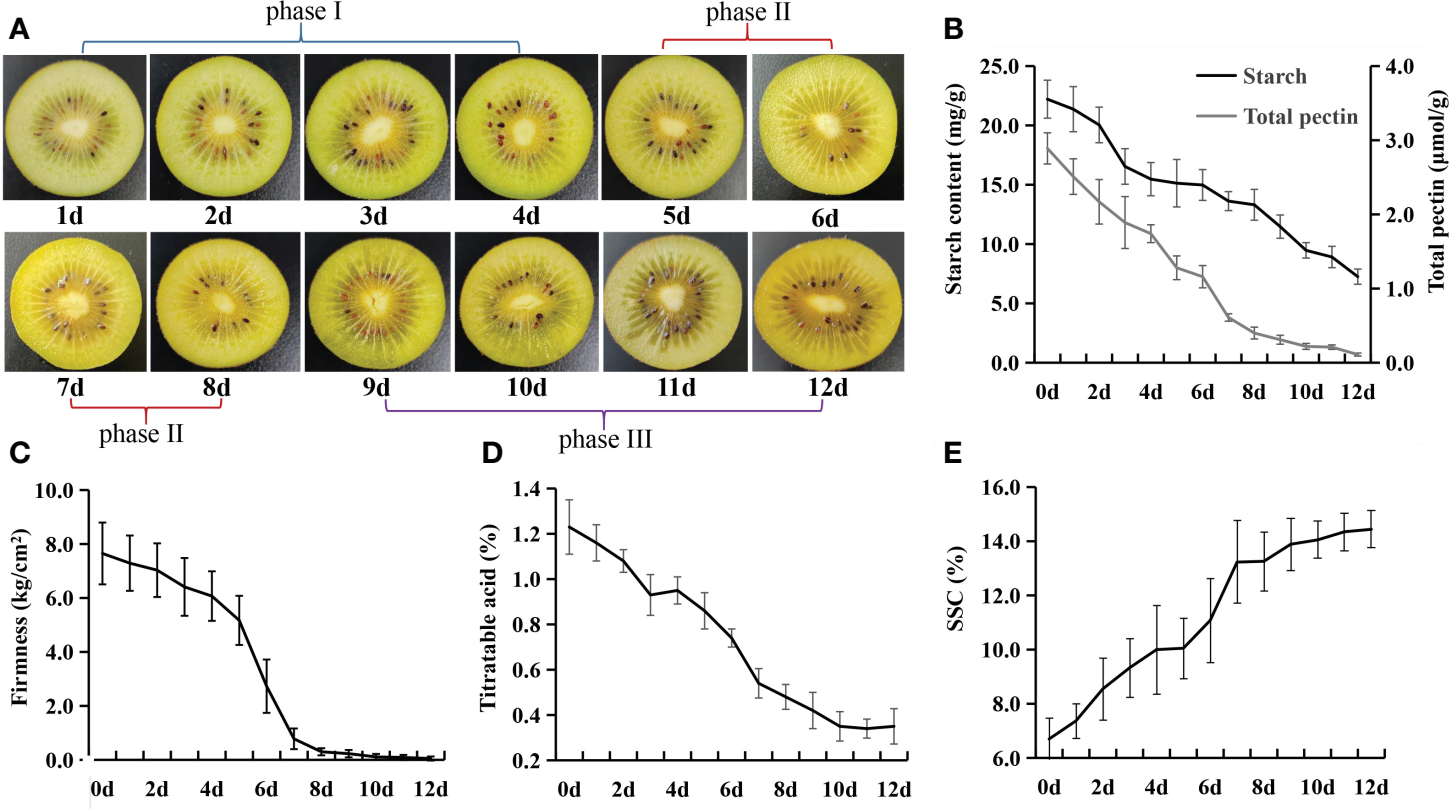
Twelve ripening stages at 20°C selected for analysis of *Actinidia chinensis* cv. Jinyan. **(A)** Color changes of flesh during postharvest ripening. **(B)** Changes in starch and total pectin content during ripening. **(C)** Changes in fruit firmness during ripening. **(D)** Changes in percentage of titratable acid during ripening. **(E)** Changes in soluble solid concentration (SSC) during ripening.

To reduce postharvest loss, the commercial practice is to harvest the kiwifruit at the physiological mature stage (180 days after pollination (DAP) for *Actinidia chinensis* cv. Jinyan). Since our objective is to uncover the complex changes in metabolites during kiwifruit postharvest ripening, the kiwifruit fruit were picked at 180 DAP and then stored at 20 °C for ripening. During the postharvest ripening, the flesh color remained pale green in phase I, and gradually changed to faint yellow from phase II to phase III (**Figure 1A**). The starch content rapidly decreased from 22.21 mg/g on 0 d to 16.54 mg/g on 3 d, and then dropped gradually from 4 to 12 d (**Figure 1B**). The total pectin content decreased rapidly in phase I and phase II, and then slowly in phase III (**Figure 1B**). The fruit firmness sharply decreased from 5 to 7 d (phase II), and reached the accepted edible ripening stage at 7 d (**Figure 1C**). The titratable acid showed gradual decreases from 0 to 10 d (**Figure 1D**). SSC sharply increased from 5 to 7 d and reached the peak of 14.4% at 12 d, which is consistent with the timing of changes in firmness (**Figure 1E**).

### Metabolism of soluble sugars and organic acids during postharvest ripening

A total of 12 soluble sugars were identified in the postharvest fruit of *Actinidia chinensis* cv. Jinyan, including nine monosaccharides and three disaccharides (**Supplementary Table 2**). The dominant soluble sugars are sucrose, fructose, glucose, inositol and galactose, while the contents of other seven soluble sugars were all lower than 0.5 mg/g (**Table 1**). Sucrose, fructose, and glucose showed similar variation tendencies in content, which gradually increased along with ripening and reached 2.53, 2.22 and 2.10 folds in the edible ripening stage relative to those in the

**Table 1:** Contents of major soluble sugars and organic acids at different ripening stages in ‘jinyan’ kiwifruit (mg/g).

Soluble sugars	0d	1d	2d	3d	4d	5d	6d	7d	8d	9d	10d	11d	12d
Sucrose	22.4± 1.8	26.0 ± 2.3	32.5 ± 2.1	49.6 ± 3.1	50.5 ± 2.6	53.4 ± 1.8	55.8 ± 3.0	56.6 ± 2.4	58.9 ± 1.5	59.5 ± 1.8	59.7 ± 3.2	59.9 ± 2.9	59.9 ± 2.1
Fructose	30.4 ± 1.7	32.4 ± 1.8	36.6 ± 1.7	39.5 ± 2.5	52.1 ± 3.1	57.6 ± 1.9	59.6 ± 2.5	67.6 ± 2.9	73.0 ± 2.9	79.3 ± 1.9	79.9 ± 2.3	80.2 ± 3.4	82.6 ± 1.6
Glucose	35.8 ± 2.4	39.2 ± 2.6	42.2 ± 3.0	46.8 ± 2.8	57.9 ± 2.3	57.9 ± 2.7	65.6 ± 3.3	75.2 ± 3.8	75.2 ± 3.5	82.8 ± 1.4	83.8 ± 3.1	84.7 ± 4.1	86.6 ± 2.1
Inositol	8.7 ± 0.3	8.7 ± 0.5	6.8 ± 0.4	7.1 ± 0.6	8.1 ± 1.1	8.2 ± 0.4	8.0 ± 1.2	9.9 ± 0.8	7.8 ± 0.5	8.7 ± 0.8	9.10 ± 0.6	8.3 ± 0.7	8.3 ± 0.8
Galactose	1.0 ± 0.2	1.0 ± 0.2	1.1 ± 0.3	0.9 ± 0.2	1.0 ± 0.2	0.9 ± 0.1	1.1 ± 0.2	1.1 ± 0.2	0.9 ± 0.1	0.8 ± 0.2	0.9 ± 0.2	0.9 ± 0.1	0.8 ± 0.2
Citric acid	10.6 ± 1.3	10.4 ± 1.3	10.3 ± 0.8	10.2 ± 1.1	9.6 ± 0.9	9.3 ± 0.4	9.3± 0.6	8.8 ± 0.7	8.7 ± 0.5	8.6 ± 0.4	8.1 ± 0.2	7.9 ± 0.4	7.5 ± 0.3
Quinic acid	9.0 ± 0.6	9.0 ± 0.6	9.1 ± 0.3	9.5 ± 0.8	9.6 ± 0.6	9.8 ± 0.4	10.4 ± 0.8	11.6 ± 0.5	10.7 ± 0.6	10.4 ± 0.6	9.4 ± 0.4	9.3 ± 0.8	9.2 ± 0.4
Malic acid	2.4 ± 0.3	2.6 ± 0.3	2.9 ± 0.2	2.8 ± 0.4	2.6 ± 0.3	3.0 ± 0.4	2.6 ± 0.5	2.6 ± 0.3	3.1 ± 0.2	2.9 ± 0.3	3.1 ± 0.5	3.0 ± 0.2	2.3 ± 0.3

physiological mature stage, respectively. However, there was no significant change in

inositol and galactose content during ripening. A total of 31 organic acids were identified in the postharvest fruit of *Actinidia chinensis* cv. Jinyan (**Supplementary Table S3**). The major organic acids included quinic acid, citric acid and malic acid, and the contents of other organic acids were all lower than 1.0 mg/g (**Table 1**). The citric acid content gradually decreased during ripening and showed a similar variation tendency to titratable acid with a correlation coefficient of 0.96. The quinic acid content gradually increased from 0 to 7 d, and then gradually decreased from 8 to 12 d. The malic acid content exhibited no significant change during ripening (**Table 1**). Cluster heatmap analysis based on organic acid accumulation levels at different ripening stages revealed that the 31 organic acids could be divided into two clades (**Figure 2**): clade I comprised a total of 17 organic acids whose overall content decreased during ripening, and clade II included 14 organic acids whose overall content increased during ripening.

**Figure 2:**
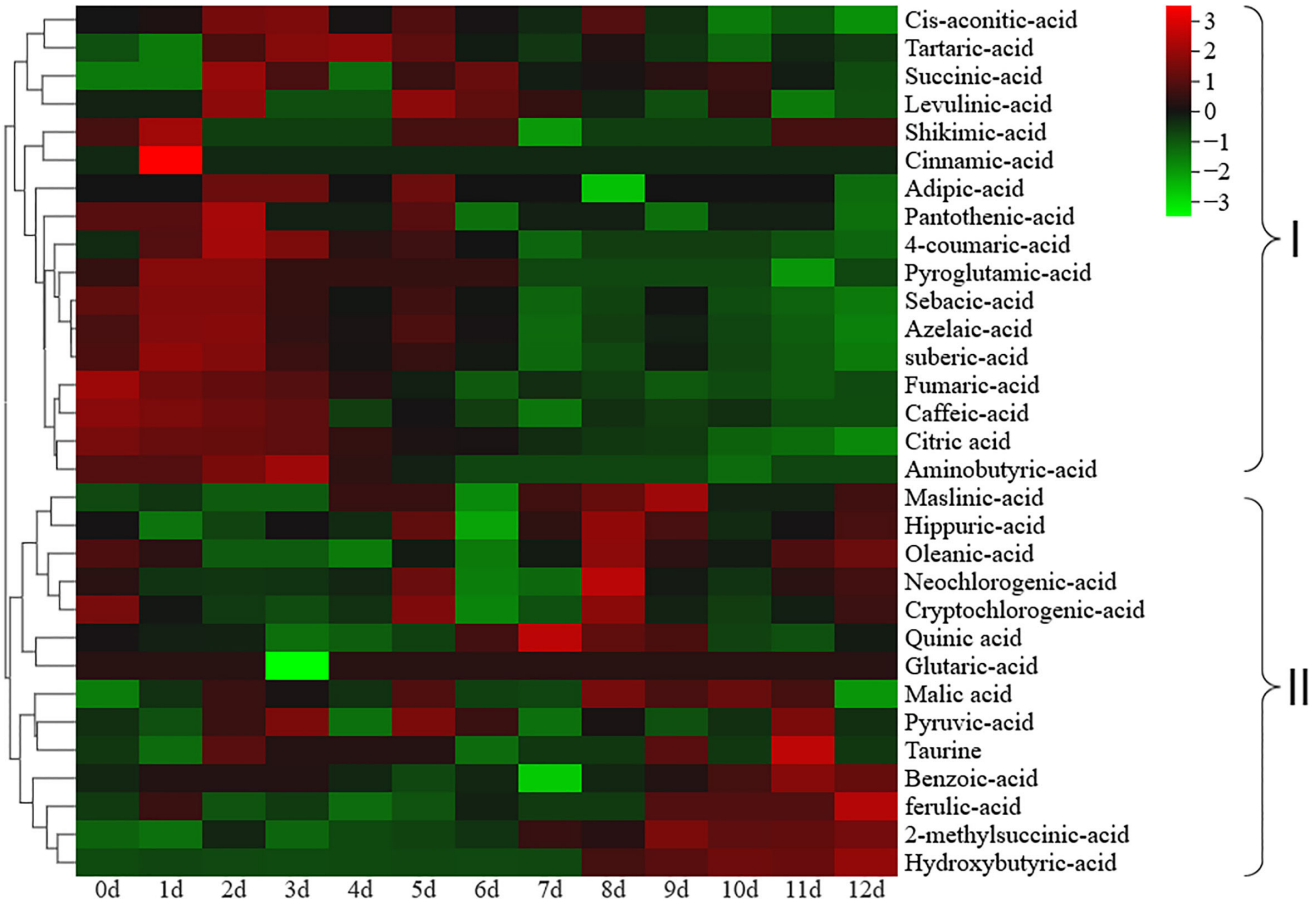
Cluster heatmap analysis based on organic acid accumulation levels at different ripening stages. Organic acids in group I gradually decreased during ripening, while organic acids in group II gradually increased during ripening.

### Energy metabolism during postharvest ripening

**Figure 3:**
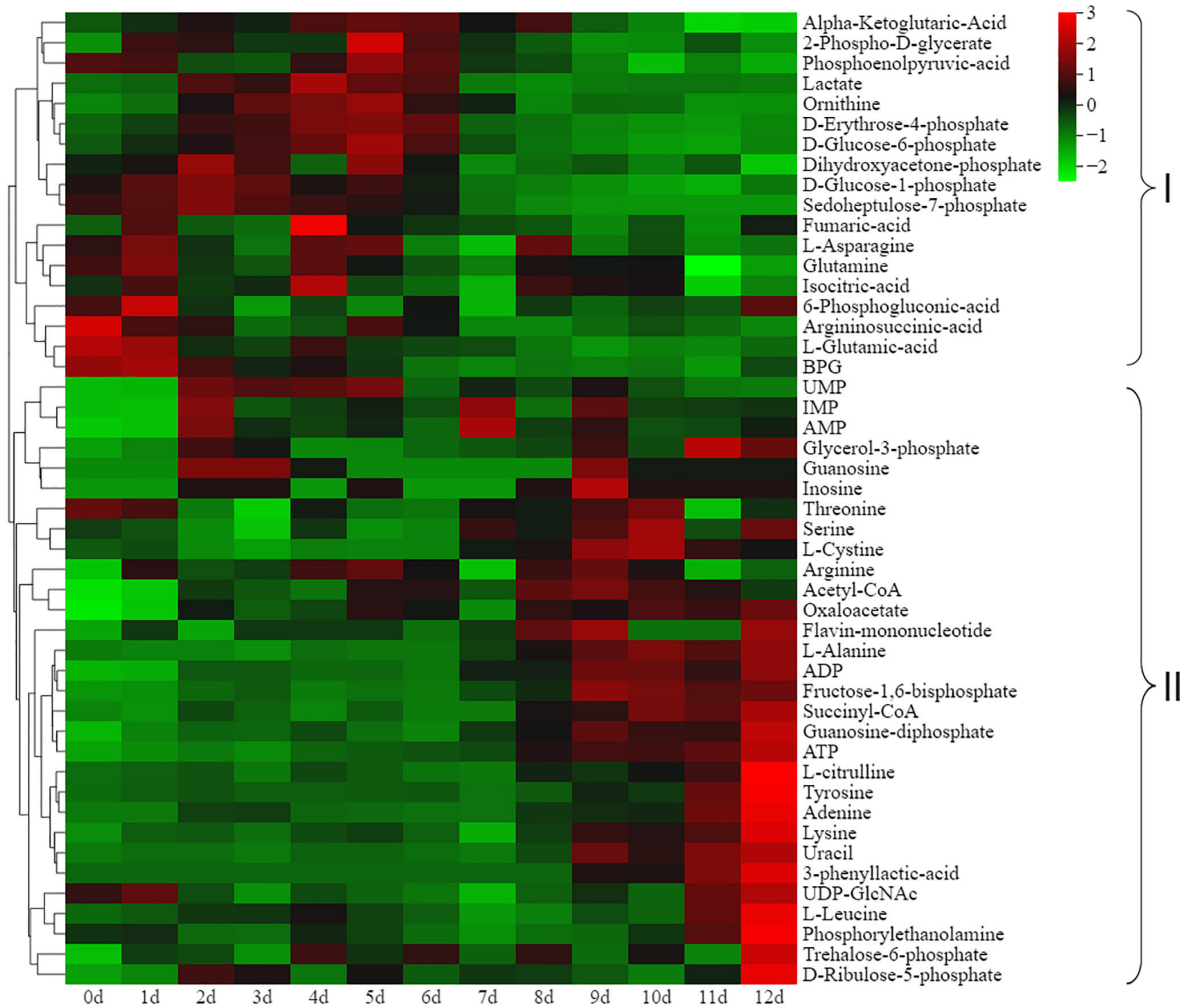
Cluster heatmap analysis based on the energy metabolite accumulation levels at different ripening stages. Organic acids in clade I and clade II gradually decreased and increased during ripening, respectively.

To evaluate the energy status of the respiratory metabolic pathway during postharvest ripening, we monitored the changes in energy metabolites by UPLC-MS/MS. A total of 48 energy metabolites were identified, including one amino acid derivative, one lysophosphatidylethanolamine, three coenzymes and vitamins, five phosphric acids, seven organic acids and their derivatives, eight phosphate sugars, 11 nucleotides and their metabolites and 12 amino acids (**Supplementary Table S4**). Cluster heatmap analysis based on energy metabolite accumulation levels at different ripening stages demonstrated that the 48 energy metabolites could also be divided into two groups (**Figure 3**). Among them, 18 energy metabolites showed significantly higher accumulation levels at the non-edible stage (0 to 6 d) than at the edible stage (7 to 12 d) (**Figure 3**, clade I), while it was the opposite case for the 30 energy metabolites in clade II (**Figure 3**, clade II). These results implied that the intensity of respiratory metabolism in kiwifruit significantly increased with fruit reaching the ripen and softening status. In addition, it is worth noting that threonine, serine, L-cystine, arginine, L-alanine, L-citrulline, tyrosine, lysine and L-leucine were more abundant in the edible stage than in the non-edible stage.

### Changes in endogenous hormones during postharvest ripening

To investigate the endogenous hormone components in kiwifruit fruit and their content variation patterns during postharvest ripening, we selected samples of seven ripening stages (0, 2, 4, 6, 8, 10 and 12) for LC-MS/MS analysis. A total of 35 endogenous hormones were identified, including one abscisic acid (ABA), one ethylene, one strigolactone, three gibberellins, six jasmonic acids, six auxins and 17 cytokinins (**Supplementary Table S5**). 1-aminocyclopropanecarboxylic acid (an average of 220.38 ng/g) was the hormone with the highest overall accumulation level, followed by ABA-glucosyl ester (an average of 36.31 ng/g) and cis-12-oxophytodi-

enoic acid (an average of 28.08 ng/g). Interestingly, the accumulation level of these three hormones showed no significant change during postharvest ripening. Nine hormones exhibited significant changes during postharvest ripening. The contents of dihydrozeatin (DZ), isopentenyl-adenine-7-glucoside (IP7G), isopentenyl-adenine-

9-glucoside (IP9G), isopentenyladenine (IP), and 2-Methylthio-cis-zeatin riboside (2MeScZR) stayed at low levels from 0 to 6 d, and showed sharp increases from 8 to 12 d. The contents of dihydrozeatin-7-glucoside (DHZ7G) and dihydrozeatin-

**Figure 4:**
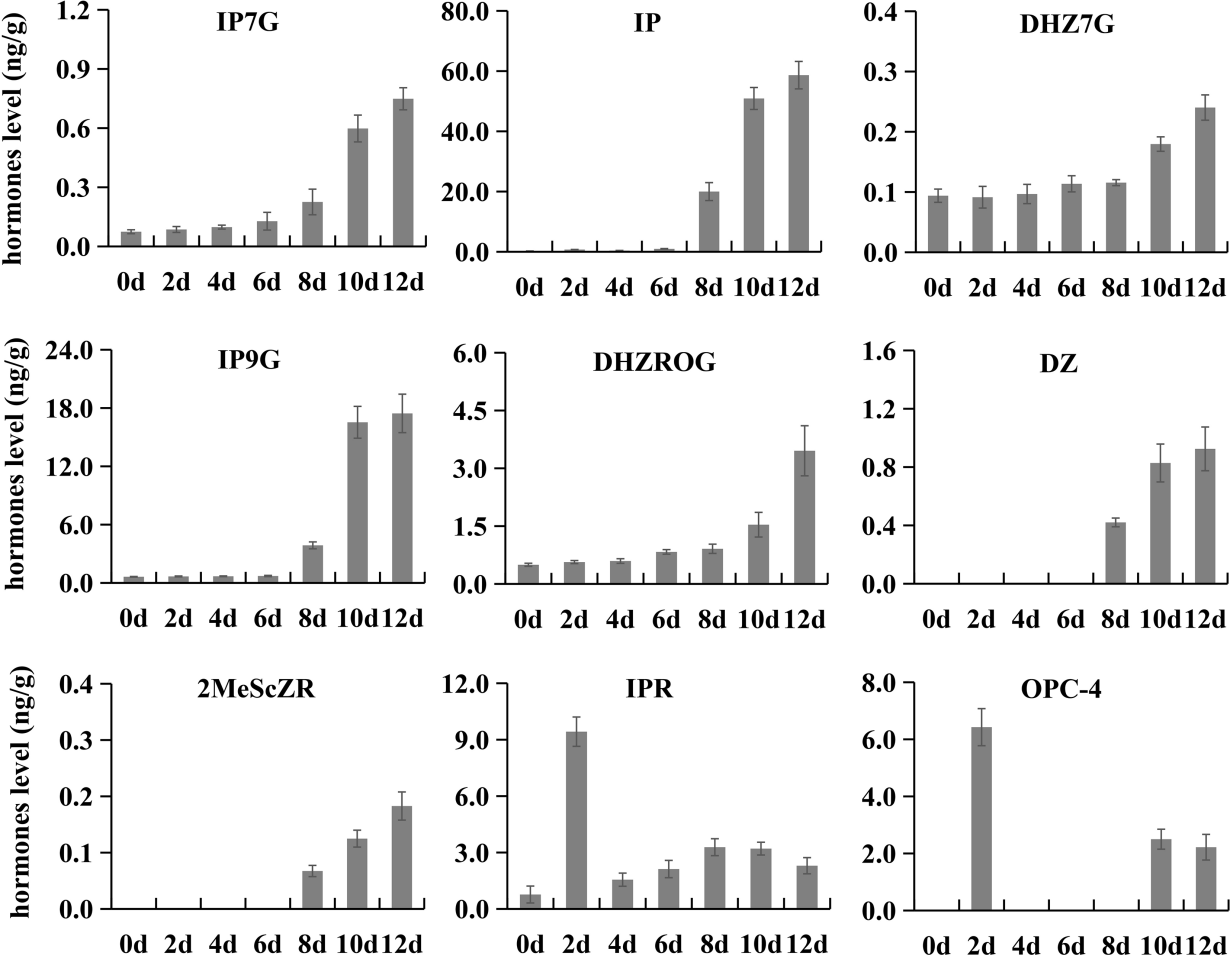
Contents of nine hormones at different postharvest ripening stages. Error bars represent standard deviations from three replicates.

10-glucoside riboside (DHZROG) gradually increased in phase I and phase II, and then rapidly grew in phase III (10 to 12 d). 3-oxo-2-(2-(Z)-Pentenyl) cyclopentane-1-butyric acid (OPC-4) and isopentenyladenosine (IPR) showed similar variation patterns in content with a particularly high accumulation at 2 d of ripening (**Figure 4**).

### Changes in enzyme activities related to sugar and organic acid metabolism and softening

**Figure 5:**
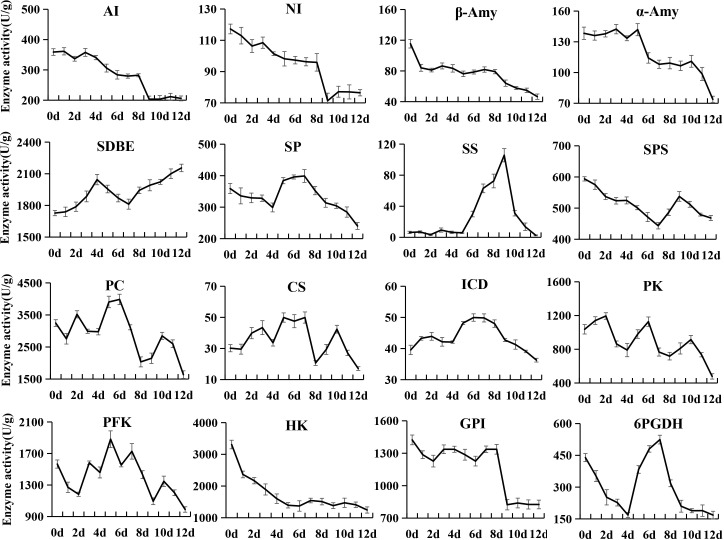
Changes in enzyme activities related to sugar and organic acid metabolism in kiwifruit during postharvest ripening. The error bars represent the SD from three replicate.

**Figure 6:**
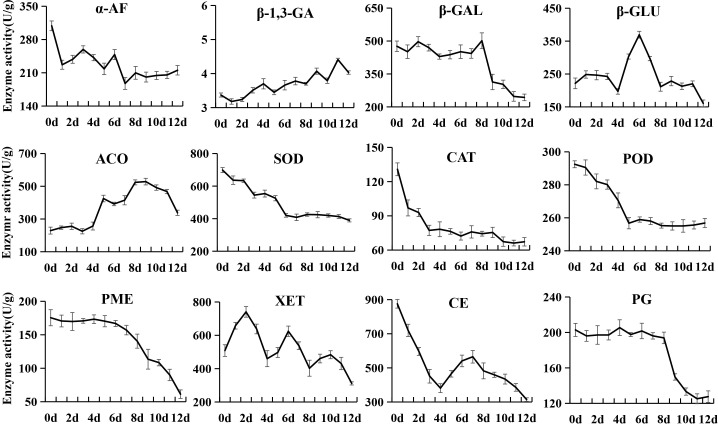
Changes in enzyme activities related to the softening of kiwifruit during postharvest ripening. The error bars represent the SD from three replicate.

During the postharvest ripening of kiwifruit, changes in the activity of 28 enzymes were analyzed, among which 16 enzymes were related to sugar and organic acid metabolism (**Figure 5**) and the remaining 12 enzymes were related to softening (**Figure 6**). AI and NI showed similar variation patterns in activity, both of which were at high levels in phase I and phase II, and then decreased rapidly in phase III. In addition, the overall activity of AI in postharvest kiwifruit fruit was 3.12-fold that of NI. The β-Amy activity sharply decreased from an initial value of 115.47 U/g to 84.07 U/g at 0 d, and then remained at this level until 8 d (phase I ~ phase II), followed by gradual decreases (phase III). The activity of α-Amy stayed at around 138.33 (U/g) from 0 to 5 d, rapidly decreased to 110.25 (U/g) at 6 d, and was then maintained at this level until 10 d, followed by a sharp decrease. The SDBE activity first increased in phase I and then fell in phase II (4 to 7 d), and finally increased again (7 to 12 d), while a completely opposite trend was observed for SP and 6-phosphogluconate dehydrogenase (6PGDH) activity. The sucrose synthase (SS) activity was stably low from 0 to 5 d (around 6.37 U/g), and then sharply increased to 105.69 at 9 d, followed by a rapid decrease. SPS activity gradually decreased from 0 to 7 d, and then increased to 537.47 U/g at 9 d, followed by a slow decrease. The pyruvic carboxylase (PC), citrate synthase (CS) and PFK activities showed an alternating pattern of decreasing and increasing, while it was the opposite for the pyruvate kinase (PK) activity. The isocitrate dehydrogenase (ICD) activity was stable (36.27~49.96 U/g) throughout ripening. The HK activity rapidly decreased from an initial value of 3309.62 U/g to 1393.40 U/g at 5 d, and then stayed stable at this level. The phosphoglucose isomerase (GPI) activity was stably high (around 1310.86 U/g) from 0 to 8 d, but decreased dramatically to 823.94 U/g at 9 d, and then remained stable until the end of storage.

The α-AF activity rapidly decreased from an initial value of 308.86 U/g to 226.89 U/g at 1 d, and was then stable throughout the ripening. The β-1,3-Glucanase (β-1,3-GA) activity always remained at very low levels (3.18~4.41 U/g) during postharvest ripening. The β-GAL, PG and PME activities were high in phase I and phase II, and then sharply declined from phase III. β-GLU activity first showed a rapid increase from 4 to 7 d, and then dramatically decreased to the initial level value at 9 d. The 1-amino-cyclopropane-1-carboxylate oxidase (ACO) activity sharply increased from 4 to 6 d, and then grew at a moderate rate and reached the peak at 9 d. The catalase (CAT), peroxidase (POD) and superoxide dismutase (SOD) activities showed similar variations, decreasing sharply to low levels at 3 d, 5 d and 6 d, respectively, and then tended to be stable. XET activity showed an alternating pattern of increasing and decreasing. The CE activity sharply decreased from an initial value of 875.57 U/g to 381.19 U/g at 4 d, and then gradually increased to 565.31 U/g at 7 d, followed by continuous decreases.

### Correlation analysis of enzyme activities and quality index of kiwifruit

Pearson’s correlation analysis demonstrated that the activities of AI, NI, SOD and POD were positively correlated with the firmness and contents of starch, total pectin, and citric acid, but negatively correlated with SSC and fructose content (**Supplementary Table 6**). On the contrary, the β-1,3-GA activity showed negative correlations with the firmness and contents of starch, total pectin and citric acid, but positive correlations with SSC and fructose content. The activities of CAT, α-Amy, and β-Amy were positively correlated with the contents of starch and total pectin. Besides, the CAT activity was negatively correlated with sucrose and SSC. The α-Amy activity showed a negative correlation with fructose and positive correlations with firmness and citric acid. The β-Amy activity was negatively correlated with SSC and fructose content, and positively correlated with citric acid. The activities β-GAL, PG and PME exhibited positive correlations with starch and citric acid content. The activities of CE, HK, and SPS were negatively correlated with sucrose content, and those of HK and PME were negatively with fructose content. A total of 11 enzymes showed no significant correlation with the quality index of kiwifruit.

## Discussion

### Changes in physiological indicators during postharvest ripening

Since soluble sugars and organic acids are key flavor components of kiwifruit, it is necessary to clarify their dynamic changes during postharvest ripening to improve the postharvest quality of kiwifruit fruit (Wang et al., 2022). In this study, 12 soluble sugars were identified in *Actinidia chinensis* cv. Jinyan (**Supplementary Table 2**). Glucose and fructose were the most important soluble sugars, followed by sucrose, which is consistent with the results of blueberry (Li et al., 2019) and watermelon (Umer et al., 2020). However, these results are inconsistent with the findings in *Actinidia chinensis* cv, Hongyang, in which the predominant soluble sugar is sucrose (Wang et al., 2022). This may be due to the different contributions of sugar import, metabolism, and dilution during the development of kiwifruit with different genotype. In addition, the difference in sugar composition is also one reason for the difference in the taste and flavor between ‘Jinyan’ and ‘Hongyang’, because sugar is also a basic raw material for the synthesis of other components such as acida, pigments, amino acids, vitamins, and aromatic substances (Dai et al., 2016). At the physiologically mature stage, the contents of fructose, glucose and sucrose were only 35.8, 30.4 and 22.4 mg/g, respectively. However, with the softening of fruit, the contents of fructose, glucose and sucrose dramatically increased to 86.6, 82.6 and 59.9 mg/g at 12 d, respectively (**Table 1**). These results further define the ripening characteristics of kiwifruit. To improve the postharvest fruit quality of kiwifruit, more studies should be carried out to explore the regulatory mechanism of soluble sugars.

The special organic acid composition and content are important reasons for the unique flavor of different fruits (Jiang et al., 2020). There are many organic acid components in fruits, but most fruits are dominated by one to three kinds of organic acids, and other components only exist in small or trace amounts (Cholet et al., 2016; Ma et al., 2018; Shi et al., 2019). In this study, we analyzed changes in the composition and content of organic acids in *Actinidia chinensis* cv. Jinyan during postharvest ripening. A total of 31 organic acids were identified, and citric acid, quinic acid, and malic acid were the dominant organic acids, which is consistent with the results reported in ‘*Hongyang*’, ‘*Zaoxian*’, ‘*Cuiyu*’, ‘*Hort16A*’, ‘*Jinyang*’, ‘*Xuxiang*’ and ‘*Cuixiang*’ of kiwifruit (Wang et al., 2017). The citric acid content gradually decreased along with fruit ripening, and showed a high correlation coefficient with titratable acid (0.96). The quinic acid content rose first and then fell, and there was no significant difference between physiological mature stage and accepted edible ripening stage (**Table 1**). In addition, the content of malic acid was stable at the level of 2.3~3.1 mg/g (**Table 1**). These results indicated that citric acid degradation is the main reason for the decrease in acidity during the postharvest ripening of kiwifruit.

### Dynamic change in energy metabolites during postharvest ripening

Respiration is the energy source of postharvest life activities, but it also plays a negative role in fruit quality maintenance. Energy status has been reported to be involved in the ripening and senescence of many horticultural crops (Zhang et al., 2019; Lin et al., 2020). In this study, a total of 48 energy metabolites were detected in *Actinidia chinensis* cv. Jinyan during postharvest ripening, mainly including Embden-Meyerhof-Parnas (EMP), tricarboxylic acid (TCA) cycle and pentose phosphate (PPP) pathway skeleton substances and their downstream products (**Figure 3**). Interestingly, when the fruit reached the accepted edible ripening stage, the accumulation levels of EMP, TCA and PPP pathway skeleton substances decreased rapidly, while there were sharp increases in the downstream products, particularly amino acids, which may explain why kiwifruit has a good taste but may rot quickly after soft ripening at the metabolic level. When the fruit reached the soft ripening stage, the respiratory intensity decreased rapidly, and the produced energy might be insufficient to maintain normal physiological functions, disease resistance and storage tolerance.

### Dynamic changes in hormones during kiwifruit ripening

Plant hormones are a kind of endogenous organic compounds with physiological activities, and play irreplaceable roles in fruit development and ripening, though their contents are generally very low (Li et al., 2014). Many exogenous hormone treatment experiments have provided new insights into the role of hormones in promoting fruit development, delaying postharvest senescence and improving post-ripening fruit quality. For instance, exogenous melatonin treatment can increase the fruit weight and sucrose and sorbitol content of peach fruit (Liu et al., 2018; Ma et al., 2021). Methyl jasmonate treatment can modulate sugar accumulation and postharvest fruit quality (Li et al., 2021). Exogenous cytokinin treatment could delay postharvest senescence of strawberry and kiwifruit (Ainalidou et al., 2016; Li et al., 2016b). However, the variation patterns of endogenous hormones during fruit postharvest ripening remain poorly understood. This study revealed the dynamic changes of 35 endogenous hormones during kiwifruit postharvest ripening. Among them, seven cytokinins (IP7G, IP, DHZ7G, IP9G, DHZROG, DZ and 2MeSeZR) (**Figure 4**) were maintained at very low levels before fruit softening, but increased rapidly when the fruit reached the edible ripening stage. We hypothesized that the rapid accumulation of cytokinins might be one factor leading to the rapid decrease of hardness in kiwifruit at the later stage of softening, though this is contrary to the experimental results of exogenous cytokinin treatment, which needs to be supported by more evidence in future studies.

### Dynamic changes in enzyme activities and correlation analysis

The softening process of kiwifruit is mainly accompanied by starch and pectin degradation, soluble sugar accumulation, organic acid degradation and cell wall disassembly, and involves a series of associated enzymes (Richardson et al., 2004; Moscatello et al., 2011; Nardozza et al., 2013). In this study, we analyzed the activities of 28 enzymes during the softening process. Among them, AI, NI, α-Amy and β-Amy showed higher activities in softening phase I and phase II, and were significantly correlated with starch, total pectin, sucrose, fructose and SSC, which is partially consistent with the results reported in previous studies (Nielsen et al., 1997; Bassinello et al., 2002). In addition, correlation analysis revealed that the activities of these enzymes were significantly positively correlated with fruit firmness. These results indicated that starch plays a supporting role in the cell and maintains its turgor pressure as an inclusion, and when starch is hydrolyzed and converted into soluble sugars and metabolized, the cell tension would decrease, thereby resulting in fruit softening (Wu et al., 2001).

PG is a key enzyme in pectin degradation, cell wall disassembly and fruit softening. It exhibited a high activity in softening phase I and phase II, which decreased in the later softening stage of ‘Jinyan’ kiwifruit. This result seems to be somewhat contradictory to the report that PG activity increased at the late stage of ‘Hayward’ ripening (Bonghi et al., 1996; Tavarini et al., 2009), but is consistent with the report of Wegrzyn and MacRae (1992). These results suggest that the enzymes related to postharvest softening have different effects in different kiwifruit cultivars. The PME activity was high in postharvest hard fruit stage (phase I) and decreased along with fruit softening, and seems to have no direct relationship with kiwifruit softening. Another possibility is that PME may change pectin molecules from high methyl ester to low methyl ester, which is more conducive to the hydrolysis of pectin by PG (Wang and Han, 1998). The β-GAL activity was stable at a high level in phase I and phase II, and rapidly decreased in phase III, which is partially consistent with previous reports (Bonghi et al., 1996; Tavarini et al., 2009).

The activities of protective enzymes such as POD, SOD and CAT that eliminate free radicals were also significantly correlated with starch, total pectin, sucrose, fructose and firmness. However, their activities reached the peak in the initial softening stage, which is contrary to the results of *Actinidia kolomikta* (Cang et al., 2001). One possible reason for this contradiction may be that with the decrease in POD, SOD and CAT activities at the later stage of softening, the ability of scavenging free radicals will decrease to accelerate the softening process. Overall, fruit softening is an extremely complex physiological process, and the changes in inclusions are definitely a result of the synergistic effects of many metabolic enzymes, while the interaction mechanism among them needs to be further studied.

## Data availability statement

The original contributions presented in the study are included in the article/**Supplementary Material**. Further inquiries can be directed to the corresponding author.

## Author contributions

XW conceived the study, participated in its design and coordination, performed the experimental measurements, processed the experimental data, interpreted the data, and drafted and revised the manuscript. JM and ZG participated in study design and coordination, performed the experimental measurements, processed the experimental data, interpreted the data, and drafted and revised the manuscript. ML, XZ and XN performed the experimental measurements and helped in sampling. XG, YL and LC processed the experimental data and helped in sampling. All authors contributed to the article and approved the submitted version.
