# Supplementary material for: Targeted multi-platform metabolome analysis and enzyme activity analysis of kiwifruit during postharvest ripening
Source: Front Plant Sci. 2023 Mar 7;14:1120166. doi: 10.3389/fpls.2023.1120166 (PMC10028114; doi:10.3389/fpls.2023.1120166)
Supplement: Supplementary file 1 [file Table_1.docx]

**Supplementary Table 1** The information of 28 enzymes assay kits

| Enzymes | Catalog Number | Producers |
| --- | --- | --- |
| β-1,3-Glucanase (β-1,3-GA) | AKSU038M | Beijing Boxbio Science & Technology Co., Ltd, China |
| β-Galactosidase (β-GAL) | AKSU042C |  |
| 6-Phosphogluconate Dehydrogenase (6PGDH) | AKCO012U |  |
| Sucrose Synthase (SS) | AKSU021M |  |
| Pyruvate Kinase (PK) | RX91019 | Ruixin Biological Technology Co. Ltd, China |
| Superoxide dismutase (SOD) | RX91021 |  |
| Polygalacturonase (PG) | RX91030 |  |
| Pectin methylesterase (PME) | RX91040 |  |
| Catalase (CAT) | RX91047 |  |
| Peroxidase (POD) | RX91048 |  |
| hexokinase (HK) | RX91058 |  |
| phosphofructokinase (PFK) | RX91066 |  |
| Citrate synthase (CS) | RX91077 |  |
| cellulase (CE) | RX91107 |  |
| Isocitrate dehydrogenase (ICD) | RX91123 |  |
| sucrose phosphate synthetase (SPS) | RX91137 |  |
| Pyruvate carboxylase (PC) | RX91180 |  |
| acid invertases (AI) | RX91284 |  |
| α-amylase (α-Amy) | RX91452 |  |
| β-glucosidase (β-GLU) | RX91550 |  |
| starch debranching enzymes (SDBE) | RX91593 |  |
| α-arabinfuranosidease (α-AF) | RX91673 |  |
| neutral invertases (NI) | RX91724 |  |
| β-amylase (β-Amy) | RX91674 |  |
| starch phosphorylase (SP) | RX930623 |  |
| xyloglucan endotransglycosylase (XET) | RX93181 |  |
| Phosphoglucose Isomerase (GPI) | RX98796 |  |
